# Assessment of outcomes in postaneurysmal subarachnoid bleed patients admitted to the intensive care unit utilizing the subarachnoid haemorrhage international trialist clinicoradiological prediction model for dichotomised functional outcome and mortality

**DOI:** 10.1016/j.ccrj.2025.100126

**Published:** 2025-10-22

**Authors:** David Mogg, James Walsham

**Affiliations:** a Intensive Care Unit, Mater Hospital, South Brisbane, Australia; b Intensive Care Unit, Princess Alexandra Hospital, South Brisbane, Australia; c Royal Flying Doctors Service (RFDS) Queensland Section, Australia

**Keywords:** Subarachnoid, Intensive care, Aneurysmal, Prediction, Model

## Abstract

**Objective:**

The objective of this study was to assess the Subarachnoid Haemorrhage International Trialist (SAHIT) prediction model in a tertiary adult intensive care unit (ICU) cohort when assessing patient outcomes against predicted outcomes, firstly by assessing the discrimination and validation of the model in the Princess Alexandra Hospital (PA) intensive care cohort and secondly comparing the predicted outcomes using the SAHIT model to the actual cohort outcomes using a Monte Carlo simulation.

**Methods:**

Six logistic regression models designed by the SAHIT Collaboration Group were applied to the PA cohort considering early predictive factors such as clinical grade and treatment modality to predict the risk of both mortality and unfavourable outcome at 6 months according to the Glasgow Outcome Score. The six SAHIT logistic regression models were applied to a retrospectively collected cohort of aneurysmal subarachnoid patients who were admitted to the ICU, generating individual risk scores for mortality and poor functional outcome. Area under the curve (AUC) and calibration slope/intercept and Brier score were used to assess the strength of the model in interpreting the current data set. A Monte Carlo analysis was used to compare the actual mortality outcomes to the predicted outcomes to determine if the cohort performance was better or worse than predicted by the mortality model.

**Results:**

Overall, the PA cohort actual mortality was higher than the predicted mortality rate based on the risk scores generated by the SAHIT models, demonstrated by Monte Carlo simulation using the SAHIT model risk scores. The core, neuroimaging, and full models for functional outcome produced AUCs of 0.719 (95% confidence interval [CI]: 0.55–0.84), 0.709 (95% CI: 0.55–0.83), and 0.738 (95% CI: 0.58–0.85). Regarding mortality, the respective AUCs were 0.684 (95% CI: 0.57–0.78), 0.678 (95% CI: 0.56–0.77), and 0.749 (95% CI: 0.64–0.84). Regarding calibration, there was modest calibration in general, with higher degrees of calibration in the fully functional outcome model.

**Conclusion:**

The cohort outcomes for mortality occurred at a rate higher than the risk predictions suggested using the logistic regression created by the SAHITs. Applying the externally trained model provided adequate discrimination and modest calibration, yet underestimated risk when applied to the intensive care cohort, reflected in the probability density function analysis. Using the SAHIT models in this cohort may result in underestimation of mortality for the individual patient, and the accuracy of the model is not sufficient for individual patient prediction. These results challenge the appropriateness of using admission-based models for dynamic ICU populations and highlight the urgent need for critical care–specific prognostic tools.

## 1 Introduction

Subarachnoid haemorrhage due to aneurysmal bleed (aSAH) is an uncommon and devastating form of cerebrovascular accident, resulting in significant mortality and morbidity.^1^ Early prediction of outcome assists resource allocation, anticipation of complications, augments family discussion regarding prognosis and expectations and allows for benchmarking of clinical performance.^2^^,^^3^ There is enormous variability in aSAH care, reflected in significantly different outcomes within the large trialist data sets that have been used to generate prediction models.^4^ The 2023 guidelines from the American Heart Association (AHA) and American Stroke Association (ASA) indicate the need for establishing grading scales to assist in prognostication, alongside the variability that can occur with patient outcome dependent on the infrastructure present within the receiving medical facility.^1^ Many prediction models suffer from a series of issues with regards to their general use within the clinical setting: lack of external validation in higher-acuity settings, inconsistent calibration metrics, with few studies reporting measures of calibration, essential for assessing model fit, alongside small sample sizes and missing data–handling issues, leading to potential overfitting or underfitting in the lower- or higher-acuity population compared to the development cohorts.^3^^,^^5^^,^^6^

The Subarachnoid Haemorrhage International Trialist (SAHIT) model has been subjected to extensive external validation when compared to contemporary subarachnoid haemorrhage (SAH) risk models and has been validated with both retrospectively and prospectively maintained disease-specific registries, with six independent studies of both randomised trials and observational population-based cohorts providing validation; however, there has not yet been an attempt to externally validate within an intensive care unit (ICU)–specific cohort.^7^^,^^8^^,^^9^ The SAHIT predictive model set was produced via analysis of the variables most strongly predictive of the outcome of mortality and poor functional outcome, defined by a dichotomised Glasgow Outcome Scale in a large repository of aSAH patients taken from both observational studies and prospective randomised controlled trials (RCTs).^1^ It has the advantages of ease of application at admission and model parsimony—deployable in relatively resource-poor settings compared with scoring systems requiring more advanced imaging techniques—and has been shown to predict long-term functional outcome, up to 2 years post ictus.^8^ It is also one of the largest trials to attempt prognostication algorithmically, exceeding 10,000 patients collated from its constituent trials.^2^ Naturally, being generated from trial cohorts, there is a bias towards lower-risk patients, who presented with lower clinical and radiological grades and had less premorbid burden than a proposed intensive care–specific cohort.^3^

The SAHIT study conducted the multivariate analyses to select the variables for use in the SAHIT model.^2^ Binary logistic regression was then conducted by the SAHITs to develop a set of both mortality and unfavourable functional outcome prediction models, where unfavourable outcome was defined as a Glasgow Outcome Score of 1 (dead) to 3 (severe disability), with a good outcome being a GOS of 4–5. Although not necessarily an optimally patient-centred metric, all current studies utilise the Glasgow Outcome Score (or the equivalent modified Rankin Scale [mRS]) to measure satisfactory outcomes.^10^^,^^11^ To analyse the outcomes of the Princess Alexandra (PA) cohort included in this study, the SAHIT regression models were recreated using R Project. The full coefficient and intercept data used by the SAHIT study can be found in the Supplemental Appendix, in Table (a).

We hypothesised that the SAHIT model would demonstrate systematic calibration drift when applied to an ICU-specific cohort, reflecting population-level differences rather than enabling individual patient prognostication. Thus, a retrospective cohort study was conducted. Of the 29 existing aSAH prediction models, none specifically look at a high-risk cohort.^3^

## 2 Methods

### 2.1 Ethical approval

The Human Research Ethics Committee of Metro South Hospital and Health Service granted approval for the study, HREC 2021/QSM/80544.

### 2.2 Study population and data collection

The study was a retrospective review of 7 years of aneurysmal subarachnoid patients admitted to a tertiary ICU. Patients were included on a retrospective basis, recruiting patients who were admitted to the PA ICU with acute angiographically confirmed aneurysmal SAH, captured from presentation using Australian and New Zealand Intensive Care Society coding, aged >18 years, with patient data gained from electronic medical records and imaging systems, over a 7-year duration from 2014 to 2021. The Australian and New Zealand Intensive Care Society codes included patients with aneurysmal subarachnoid bleeds admitted before or after intervention and those presenting with arteriovenous malformations, who were excluded upon manual review to limit inclusion to saccular aneurysmal subarachnoid patients categorised based on discrete size and location of the aneurysm(s). The ICU is a 30-bed unit which treats a mix of adult neurosurgical, cardiothoracic, trauma, and liver transplant patients, among more general intensive care patients, and functions alongside a well-staffed neurosurgical high-dependency unit (HDU) who typically take aSAH patients who present with lower-grade aSAH and do not require higher levels of organ support, such as mechanical ventilation or vasopressors (see Table 2, Table 3).

Data collection took place via a single independent researcher using patient electronic data records. Data for individual patients were collected based on the variables required by the SAHIT models. Follow-up data for the real mortality and functional outcomes were obtained via retrospective assessment of patient records determining the binary outcome of 12-month mortality and the functional outcome, via application of the Glasgow Outcome Scale (GOS) at up to 12 months, in keeping with the 2-, 6-, or 12-month range for the SAHIT development cohort (conceding this varied between validation and development cohorts in the SAHIT study). Premorbid hypertension was recorded if the patient had a documented diagnosis or antihypertensive medication on chart review. Mortality was assessed up to 12 months post rupture via hospital records. Functional outcome (GOS) was taken at the latest available follow-up between 2 months and 12 months (median: 9 months, IQR: 6–12). An attempt to maintain consistency was made via use of objective measures of function, through finding variations of phrases such as “returning to work”, “independent for activities of daily living”, or “full daily support with showering and feeding” that correspond to the GOS, as detailed in Table 4 (decision matrix). Thirty sentinel cases were evaluated by two ICU registrars, with >90 % agreement. We did not formally calculate Cohen’s κ, but discrepancies were rare and were reconciled by consensus. The functional outcome data were gleaned from neurosurgical outpatient notes or rehabilitation and physiotherapy outpatient notes. A consistent rehabilitation data set formally documenting functional outcome via the GOS of mRS did not exist for these patients. Poor functional outcome was dichotomised with good outcomes and defined as a GOS of 1–3 (dead, vegetative, or severe disability). “Good outcome” was GOS 4–5. GOS was the outcome measure used by the SAHIT group to develop the model. Although the Glasgow Outcome Score (GOS) is a more limited ordinal measure than the Extended GOS, we used the standard GOS to allow consistency with the SAHIT derivation and validation cohorts.

The Fisher grade was first estimated independently by two ICU registrars and correlated with the radiology report, which was validated each time by a consultant radiologist. The World Federation of Neurosurgical Societies Score (WFNS) was a consistently documented characteristic from the first contact with emergency and neurosurgical review. With regards to those admitted from the HDU or ward, qualitative information was taken from clinical notes regarding their context for admission. WFNS grade 1–2 patients with a Fisher grade ≤3 and no organ support requirements were admitted directly to the high dependency unit (HDU) and not captured. The 40 WFNS grade 1 patients included in the cohort had requirements for invasive ventilation, early management of hydrocephalus, or need for vasoactive medications. If a patient was readmitted to the ICU during the same hospital stay (for example, after surgery or for complications such as vasospasm), all data were linked to the index admission. Each patient is represented only once in our analysis; outcome (mortality and functional status) reflects their final hospital discharge. WFNS was assigned from the first documented GCS and motor deficit as follows: if sedated/intubated, the last presedation GCS from the emergency department or ambulance record was used; if the neurosurgical and emergency department GCS disagreed, the lower (worse) score was chosen.

The SAHIT models were applied to the ICU cohort as a retrospective analysis of patient outcomes to compare the performance of the study cohort to the predicted outcomes. The SAHIT models generate a risk score for both poor functional outcome and mortality using the progressively more detailed data to apply to the core, neuroimaging, or full model: each subsequent model from “core” through to “full” adds more clinical and radiological data points as it appears along the course of a patient’s journey. Subsequently, a Monte Carlo simulation was performed using the risk scores generated from the PA cohort to determine the predicted mortality. A probability density function was created from simulated data projected from the individual risk prediction scores of the cohort.

### 2.3 Model application

Model application and data generation was conducted using R. Model discrimination was evaluated using the receiver operator characteristic curves and calibration via a collation of metrics including the calibration slope and Brier score. The PAH cohort data were processed through the six SAHIT models, including the three for mortality (core, neuroimaging, and full) and the three for functional outcome (core, neuroimaging, and full), henceforth referred to collectively as the SAHIT “model”.

### 2.4 Performance assessment

To benchmark the cohort mortality and poor functional outcome against the SAHIT cohort, a Monte Carlo simulation was conducted, where the risk of death prediction facilitated by the original SAHIT logistic regression model was used to attribute mortality risk to each patient in the study cohort and subsequently generate a probability distribution function using a large simulated cohort of 10,000 patient simulations with a 95% confidence interval (CI), to compare the observed event rate to the predicted (both binary outcomes of mortality and poor functional outcome).

The model performance and discrimination were tested using the receiver operating characteristic analysis by measuring the area under the curve (AUC) as the primary measure. An AUC of 0.5 would indicate no discrimination, whereas an AUC >0.7 would be acceptable, although typically, an AUC >0.8 would be ideal.^5^^,^^12^ A calibration interception indicating perfect calibration = 0, whereas the slope of the calibration curve would be 1 for a perfectly calibrated model. The AUC was utilised to quantify how well the model could discriminate between high and low risk of poor functional outcome and mortality, in keeping with the SAHIT study. A Brier score indicated accuracy measure (range: 0–1, lower is better), with values <0.25 indicating useful predictions. The calibration slope was included to reflect prediction spread (<1 indicating overfitting) to compare to the validated SAHIT studies to indicate model calibration, in accordance with *Transparent Reporting of a multivariable prediction model for Individual Prognosis or Diagnosis* reporting standards.^13^ Calibration intercept indicates whether, on average, predicted probabilities are systematically too low (intercept >0) or too high (intercept <0). Statistical analysis was conducted using R (R Core Team, 2023, R: A language and environment for statistical computing; R Foundation for Statistical Computing, Vienna, Austria. http://www.R-project.org/). The Monte Carlo simulation followed the approach described in detail in the LOw Volume ECMO Results (LOVERS) study.^9^

Our sample size has an underpowered event rate per “rule of thumb” for external validation studies; however, it is a sequential real-world 6-year accumulation of aneurysmal subarachnoid patients requiring ICU admission.^14^

## 3 Results

### 3.1 Main results

A total of 151 patients with angiographically confirmed aneurysmal subarachnoid haemorrhage met the inclusion criteria and were included in the cohort for validation of the SAHIT predictive models. Patients lost to follow-up consisted of those discharged interstate for rehabilitation and were excluded from the analysis. The base demographics are illustrated in Table 1. Compared to the SAHIT cohort, the study cohort was of a similar age (55 years vs SAHIT 53 years) but exhibited marked differences in the proportion of patients presenting with Fisher grade 4 SAH (75% vs SAHIT 20%) and those presenting with a poor neurological grade of WFNS 4–5 (47% vs SAHIT 29%). The anatomical location showed a small degree of variation, primarily in the proportion of posterior circulation aneurysms (34% in PA vs SAHIT 12%). More patients had premorbid hypertension in the PA cohort.

**Table 1:** Patient characteristics table compared with the SAHIT cohort.

Baseline characteristics		
	PA cohort	SAHIT development cohort
Variables		
N	151	10936
**Average age (** ± **SD)**	56 years (±13)	53 (±9)
**Hypertension**		
Yes	83 (55)	2725 (37)
**WFNS grade**		
1	40 (26)	5088 (47)
2	26 (17)	2711 (25)
3	15 (10)	774 (7)
4	32 (21)	1222 (11)
5	39 (26)	1039 (10)
**Fisher grade**		
1	4 (3)	786 (8)
2	9 (6)	1635 (17)
3	25 (16)	5226 (55)
4	114 (75)	1909 (20)
**Location**		
Anterior cerebral artery	55 (36)	3469 (38)
Internal carotid artery	11 (7)	2834 (31)
Middle cerebral artery	35 (23)	1708 (19)
Posterior circulation	51 (34)	1033 (12)
**Size**		
0–12 mm	135 (88)	7328 (79)
13–24 mm	16 (11)	1337 (15)
>25 mm	1 (1)	566 (6)
**Treatment**		
Surgical	47 (31)	7497 (68)
Endovascular	81 (53)	2503 (23)
Conservative	24 (16)	936 (9)
**Mortality (12 months)**	**(Up to 12 months)**	**(From 2 to 12 months)**
	40 (26)	1317 (13)
**Poor functional outcome (12 months)**	**(Up to 12 months)**	**(From 2 to 12 months)**
	57 (37)	2951 (29)

SAHIT: subarachnoid Haemorrhage International Trialist; SD: standard deviation.

**Table 2:** Comparison of the predictive strength of the logistic regression models between PA and SAHIT cohorts.

	Unfavourable Outcome		Mortality	
	PA	SAHIT	PA	SAHIT
**Core model**	0.72 (0.55–0.84)	0.76 (0.71–0.81)	0.68 (0.57–0.78)	0.74 (0.69–0.79)
**Neuroimaging**	0.72 (0.55–0.83)	0.77 (0.72–0.81)	0.68 (0.56–0.77)	0.75 (0.72–0.79)
**Full model**	0.74 (0.58–0.85)	0.80 (0.75–0.85)	0.75 (0.64–0.84)	0.77 (0.73–0.80)

SAHIT: subarachnoid Haemorrhage International Trialist.

**Table 3:** Mortality outcome and functional outcome sensitivity analysis—model performance excluding patients with conservative management (n = 127).

Model (Mortality Outcome)	AUC (95% CI)	Calibration intercept (95% CI)	Calibration slope (95% CI)	Brier score
**Full cohort (n = 151)**				
**Core**	0.68 (0.57–0.78)	0.44 (0.21–0.67)	0.84 (0.68–1.00)	0.180
**Neuroimaging**	0.68 (0.56–0.77)	0.46 (0.23–0.69)	0.82 (0.66–0.98)	0.182
**Full**	0.75 (0.64–0.84)	0.44 (0.21–0.67)	0.84 (0.68–1.00)	0.175
**Sensitivity analysis (n = 127)**				
**Core**	0.71 (0.59–0.81)	0.18 (−0.05–0.41)	0.91 (0.72–1.10)	0.132
**Neuroimaging**	0.70 (0.58–0.80)	0.22 (−0.02–0.46)	0.88 (0.69–1.07)	0.134
**Full**	0.73 (0.61–0.83)	0.15 (−0.08–0.38)	0.93 (0.75–1.11)	0.128

Model (Functional Outcome)	AUC (95% CI)	Calibration intercept (95% CI)	Calibration slope (95% CI)	Brier score
**Full cohort (n = 151)**				
**Core**	0.72 (0.55–0.84)	−0.20 (−0.43–0.03)	1.08 (0.90–1.26)	0.215
**Neuroimaging**	0.72 (0.55–0.83)	−0.17 (−0.40–0.06)	1.05 (0.87–1.23)	0.217
**Full**	0.74 (0.58–0.85)	−0.23 (−0.46–0.00)	1.10 (0.92–1.28)	0.210
**Sensitivity analysis (n = 127)**				
**Core**	0.74 (0.58–0.87)	−0.12 (−0.35–0.11)	1.02 (0.84–1.20)	0.187
**Neuroimaging**	0.73 (0.57–0.86)	−0.08 (−0.31–0.15)	0.98 (0.80–1.16)	0.189
**Full**	0.76 (0.60–0.88)	−0.15 (−0.38–0.08)	1.04 (0.86–1.22)	0.182

AUC: area under the curve; CI: confidence interval.

**Table 4:** Decision matrix for retrospective application of GOS to electronic patient data.

GOS	Descriptor	Representative clinical phrases/indicators
**1: Death**	Death from any cause	*“Patient deceased”, “Passed away”, “Expired in ICU”, “Palliative care initiated”*
**2: Persistent vegetative state**	No awareness of surroundings, wakefulness without cognition	*“No purposeful response”, “Remains unresponsive”, “Vegetative state”, “Withdrawn treatment, remains alive”*
**3: Severe disability**	Conscious but dependent; needs assistance with basic activities of dailing living (ADLs)	*“Requires full-time care”, “Dependent for toileting and feeding”, “Severely impaired mobility”, “Transferred to high-level care facility”, “Bedbound withGlasgow Coma Scale (GCS)improvement”*
**4: Moderate disability**	Independent but unable to resume all pre-aSAH activities	*“Independent with support”, “Unable to return to work”, “Lives at home with assistance”, “Limited mobility but ambulant”, “Uses walking aid”, “Mild cognitive deficits noted”*
**5: Good recovery**	Returns to pre-aSAH life with minimal to no deficits	*“Returned to work/school”, “Independent in all ADLs”, “No functional limitations”, “Normal neurological exam”, “Living independently”*

aSAH: subarachnoid haemorrhage due to aneurysmal bleed; ICU: intensive care unit.

**Fig. 1:**
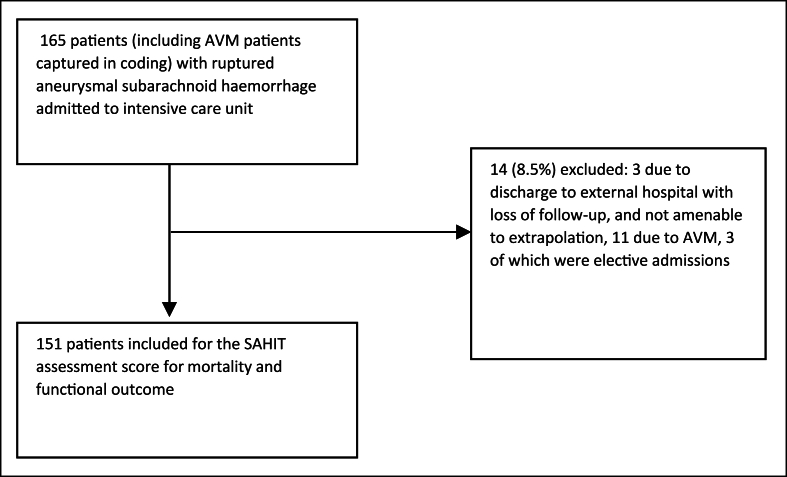
CONSORT patient selection flow diagram, depicting the number of excluded patients on the basis of inadequate/missing documentation following discharge out of state/country, or ANZICS coding capturing AVM patients. ANZICS: Australian and New Zealand Intensive Care Society; AVM: arteriovenous malformation; CONSORT: Consolidated Standards of Reporting Trials.

The SAHIT models fitted to the PA cohort show an AUC ranging from 0.68 for the neuroimaging and core mortality outcome model to 0.76 for the complete mortality model using the current cohort data, with CIs that approach 0.5. Fig. 3 shows all the reciever operator characteristic curves (ROC) curves for the core, neuroimaging, and full SAHIT models in predicting mortality (left panels) and poor functional outcome (right panels). The full-model AUC for mortality was 0.75 (95% CI: 0.64–0.84) and for poor outcome was 0.74 (95% CI: 0.58–0.85). The calibration plots in Fig. 4 demonstrate that the full models systematically underestimate mortality at higher predicted probabilities (intercept: 0.44, slope: 0.85), whereas for functional outcome, calibration was closer to ideal (intercept: 0.12, slope: 0.92) Exclusion of the conservatively managed patients in a sensitivity analysis improved calibration, but discrimination remained suboptimal: 16% received “conservative” therapy; after neurosurgical and intensivist evaluation, these patients were judged not to be candidates for aneurysm-securing intervention. They received medical management only (e.g., blood pressure control, nimodipine, and ICU monitoring) (see Fig. 1).

**Fig. 2:**
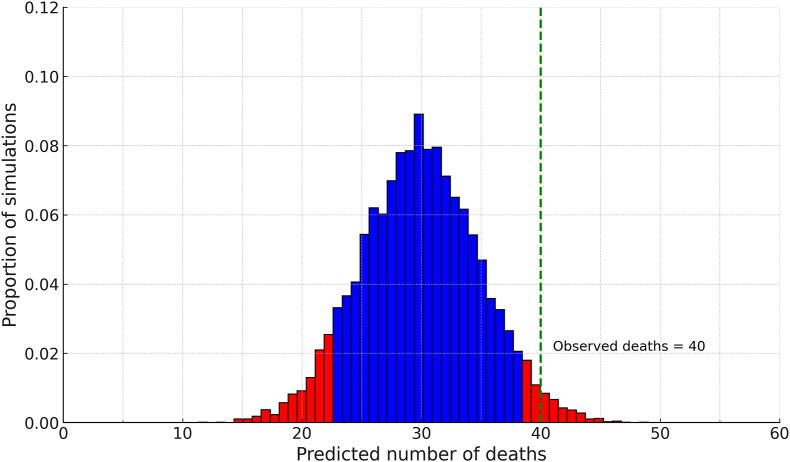
A probability distribution function using the predicted risk outcome based on the original β coefficients from SAHIT model to simulate the distribution of the number of deaths, showing the observed deaths (40, 26%) in the study cohort lies outside the 95% confidence interval for the predicted outcomes (red indicates probabilities that occur outside the 95%, expected deaths in the sample size ranging from 22 to 37).

**Fig. 3:**
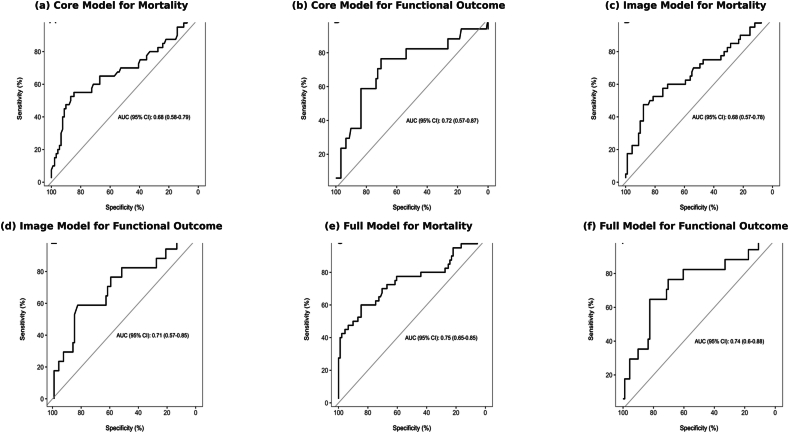
ROC plots for each model set in both binary outcomes demonstrating adequate discriminatory ability of the model when using the SAHIT logistic regression model. AUC: area under the curve; CI: confidence interval; SAHIT: subarachnoid Haemorrhage International Trialist.

**Fig. 4:**
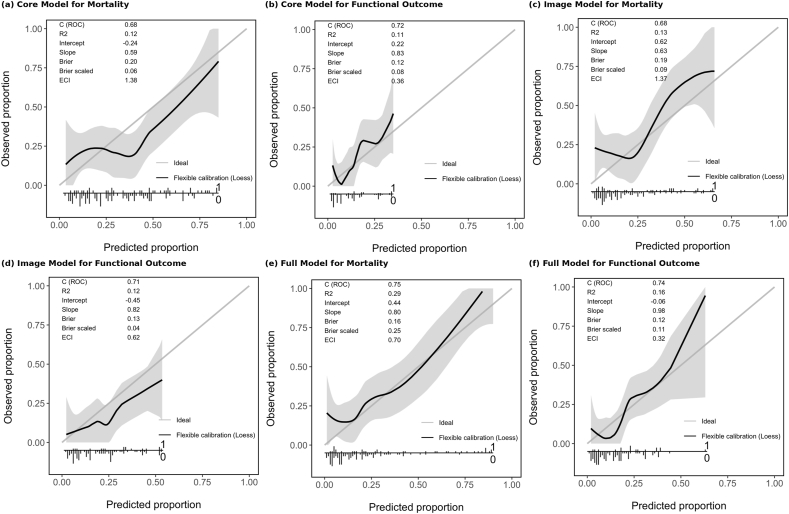
Calibration plots for each model set in both binary outcomes demonstrating mild overfitting when using the SAHIT logistic regression model. In each instance the Brier score is <0.2. SAHIT: subarachnoid Haemorrhage International Trialist. ECI: estimated calibraiton index

### 3.2 Probabilistic analysis

Using the random sampling method of modified Monte Carlo simulation, the mortality outcomes were analysed in the form of a probability density function to ascertain if observed results fell within the expected outcome. This demonstrated the cohort performed slightly worse than predicted by the SAHIT logistic regression model for mortality, as seen in the distribution present in Fig. 2, with the observed 40 deaths within the cohort of this size occurring above the 95% CI for the expected mortality distribution (22–37 expected deaths 95% CI). The Monte Carlo simulation was run to generate the probability distribution for the predicted mortality to create a comparison of the entire cohort, accounting for the small cohort size compared to large repositories such as the SAHIT cohort. In Fig. 2, the observed mortality rate fell outside of the 95% CI of the simulated distribution of deaths created using the original β-coefficients from the SAHIT study.

## 4 Discussion

Predictive scoring systems are increasingly better validated for use in aSAH, but variability in both mortality and functional outcome is prevalent, and there is significant difficulty in creating models which relate temporal causality along the patient’s hospital journey.^3^ In this single-centre ICU cohort of 151 aneurysmal SAH patients, the externally derived SAHIT risk model consistently underestimated observed mortality and showed only modest discrimination for poor functional outcome, underestimating outcomes (Fig. 2). These discrepancies were most pronounced among patients managed without aneurysm-securing intervention (“conservative” therapy), underscoring that high-acuity, ICU-only populations differ substantially from the broader SAHIT development cohorts. Clinicians should therefore be cautious when applying SAHIT predictions unadjusted to inform individual prognoses in similar settings. Although the SAHIT models have been externally validated in several cohorts and were generated from both RCTs and retrospective cohort studies, including low-grade or clinically less severe subarachnoid aneurysmal patients, this is the first time that the SAHIT model has been applied specifically to an intensive care cohort.^1^^,^^11^ The results would indicate either that the outcomes for the aSAH patients were substandard per the risk scores generated by the SAHIT model or more likely that the model does not perform as it should in this population.

The degree of discrimination is comparable to a similarly sized cohort which exhibited lower-clinical-grade aSAH patients taken from a trial registry.^7^ The sensitivity analysis demonstrates the SAHIT model’s modest inability to discriminate between good and poor outcomes in ICU patients is not primarily due to conservative treatment decisions. Even among aggressively treated patients, the model cannot reliably distinguish who will do well versus poorly. This suggests missing variables specific to ICU populations (early physiological instability, organ support requirements, and secondary complications). With regards to calibration, as seen in Fig. 4, the models showed systematic underestimation of risk. The calibration slope <1.0 indicates overfitting—the model makes overconfident predictions that diverge from reality in our sicker cohort.^5^^,^^12^ The Brier score however indicates reasonably accurate probabilistic predictions, especially in the “full” model. The full functional outcome model showed better calibration with the PA cohort with less systematic bias and scaling between predicted and actual risk. Regarding the sensitivity analysis, the improvement in calibration intercept from 0.44 to 0.18 suggests that a significant proportion of the calibration drift was attributable to patients receiving conservative management. However, the persistence of positive intercept values indicates the SAHIT model still underestimates risk even in actively treated patients. This finding validates that treatment limitation decisions were clinically appropriate rather than premature.

Comparing our cohort to the Barrow Ruptured Aneurym Trial (BRAT) trial registry reveals important distinctions^15^ (Mascitelli). Our ICU patients demonstrated higher clinical grades despite similar radiological findings—a discordance characterising ICU populations where physiological instability drives admission regardless of imaging severity. This pattern likely explains model failure as SAHIT relies heavily on radiological grading that may not capture systemic physiological derangement.^16^

Regarding treatment modality, despite 68% of our cohort receiving less invasive endovascular treatment than predominantly surgical management in SAHIT development cohorts, outcomes remained worse than predicted.^16^ This suggests treatment modality matters less than unmeasured ICU-specific factors including time to aneurysm securing, periprocedural complications, and postintervention vasospasm management.^16^ The proportion of patients exhibiting Fisher grade 3 bleeds was less than that in the SAHIT cohort, despite the volume of blood being a predictor for cerebral complications.^11^^,^^16^ Potentially as a function of higher Fisher grading or complications outside of purely neurological sequelae suffered by the PA cohort, the functional outcomes and mortality predicted were worse than expected using the SAHIT prediction model (Fig. 2.). A final selection factor that is not accounted for is the relatively small proportion of patients who initially were admitted to the HDU then deteriorated and were admitted to the ICU. It could be assumed these patients would skew mortality towards worsening outcomes; however, this was not investigated within the limits of this study and represents a limitation of the findings.

The actual mortality outcome found in this cohort occurred outside of the SAHIT-generated predicted rate of occurrence, as demonstrated in the probability density function created from the Monte Carlo simulation which was generated using the predicted risk of mortality and poor functional outcome for the PA cohort, in Fig. 2 (depicting the mortality probability density function, expected deaths: 22–37 95% CI). The sensitivity analysis demonstrates that excluding conservatively managed patients improves calibration. This could suggest two nonexclusive explanations: first, conservative treatment decisions in the study cohort appropriately reflected disease severity rather than creating self-fulfilling prophecies. Second, the SAHIT models fail to capture ICU-specific risk factors beyond treatment limitations, such as secondary complications, organ support requirements, and critical care resource utilisation.

### 4.1 Strengths and limitations

Numerous factors influence a model’s ability to generate precise predictions, and model performance deteriorates when applied outside the original development cohort.^6^ Our study cohort demonstrated higher clinical and radiological grades at presentation than the SAHIT cohort, reflecting our intentional selection of an intensive care setting—a characteristic absent from the SAHIT pooled cohort. Heterogeneity between populations regarding patient characteristics, management, and treatment approaches creates conditions that reduce calibration performance and limit clinical utility, even for models with good discrimination.^12^ Several factors may explain the degraded discrimination and calibration observed in the disparate actual outcome contrasted to the probability density functions (Fig. 2): shifts in key variables including radiological grade, higher case severity with increased unmeasured complications, temporal variations in care delivery, and differences in outcome definitions.^12^

Model performance was further compromised by variables and outcome definitions subject to interobserver variability, such as the Fisher score, and by our retrospective application of GOS to nonstandardised functional assessments. While mortality is a binary outcome, retrospectively deriving outcome scores from inconsistent datasets containing subjective data points—typically gleaned from outpatient review notes—represents a major study limitation that could only be addressed through prospectively maintained datasets or standardised review protocols for post-aSAH patients incorporating GOS or mRS. Recent imputation methods have shown reproducible and valid results in similar settings.^17^ Our retrospective GOS assignment from clinical notes, while achieving reasonable inter-rater reliability, cannot match the validity of prospective structured interviews and may have contributed to model underperformance. Regarding mortality data, mortality ascertainment was based solely on hospital records; no linkage to national registries was performed, which may limit complete capture of postdischarge deaths and introduce potential misclassification.

SAHIT’s development in trial populations with inherent selection bias limits generalisability to consecutive ICU cohorts.^2^ Dynamic models updating predictions based on complications would better serve ICU populations. For clinical utility, the SAHIT model’s reliance on admission variables limits its use primarily to benchmarking or research enrichment.^3^ Generally, acceptable model performance for individual patient outcome prediction requires an AUC exceeding 0.8–0.85, whereas models intended for population risk stratification and epidemiological assessment may be considered robust with an AUC >0.7.^9^ Consequently, although proposed as an individual risk assessment tool, the SAHIT model’s utility remains limited.^1^ A calibration intercept of 0.44 (Fig. 4) approximates a 55% relative underestimation of risk, which is clinically meaningful.

Attrition in our cohort was low (1.9%). These patients had survived to ward-level care, suggesting their outcomes likely skewed towards better functional recovery. Given our cohort’s worse-than-predicted outcomes, however, this attrition likely introduced minimal bias. The exclusion of lower-grade aSAH patients managed in the neurosurgical HDU rather than the ICU represents another limitation. Similarly, HDU-to-ICU transfers may have skewed outcomes towards worse-than-predicted results—we excluded low-grade SAH in the HDU, potentially enriching for severity yet found poor model performance. Nevertheless, all patients presenting to the ICU during the study period were included regardless of severity or aneurysm location. We acknowledge that a formal sensitivity analysis was not conducted.

Despite these data collection limitations, our cohort represents a realistic sample of consecutive, unselected patients presenting to an ICU, thus providing authentic evidence of SAHIT regression model performance for predicting mortality and functional outcomes in aneurysmal subarachnoid haemorrhage patients. This retrospective, single-centre Australian study includes a relatively small cohort compared to previous external validation efforts. And despite poor absolute calibration, SAHIT maintains relative discrimination—patients with higher scores had worse outcomes, supporting its use for cohort stratification if not individual prediction. Patients were treated within a health system with robust rehabilitation services but lacking systematised functional status documentation following intensive care discharge. Patients meeting diagnostic criteria who died shortly after admission were retained in the analysis. In contrast, much of the SAHIT data came from RCTs not originally designed for prognostic modelling, potentially introducing selection bias through trial-specific enrolment criteria. Crucially, a widely used and extensively externally validated prognostic model failed in the populations that need them the most.

Reported 30-day mortality rates in aSAH vary widely depending on illness severity and institutional context. A recent international review estimated overall 30-day case fatality at approximately 21.9%, with modest year-on-year improvement.^12^ In contrast, Scibilia et al. reported a 30-day mortality of 21% among treated WFNS grade 4–5 patients, consistent with historical rates ranging from 26–43% for poor-grade aSAH.^18^ They noted that delayed cerebral ischaemia and early treatment limitation significantly influenced short-term outcomes. Similarly, a ventilated ICU cohort in France showed 30-day mortality as high as 36%.^19^ Contextually, our cohort’s observed mortality of 26% (measured at 2, 6, and 12 months), while higher than the SAHIT derivation cohort, remains within published ranges for high-acuity populations. The over-representation of WFNS grades 4–5 (47%) and posterior circulation aneurysms (34%) in our cohort likely contributes to the elevated risk, rather than reflecting institutional underperformance. Nonetheless, unmeasured variables such as goal-of-care decisions and procedural delays may also influence these outcomes and warrant acknowledgement.

We performed the sensitivity analysis excluding conservatively managed patients. However, we acknowledge this approach has limitations since treatment modality is an included predictor in SAHIT and excluding these patients may introduce selection bias. The differential performance between the full cohort and the treated-only subset suggests SAHIT may not adequately model the interaction between baseline severity and treatment selection in ICU populations.

For practicing intensivists, these findings have immediate implications: a patient with a SAHIT-predicted mortality of 20% may face actual mortality risk approaching 30% in the ICU setting. This calibration drift reflects not model failure but rather fundamental differences between trial-enriched development cohorts and real-world ICU populations characterised by higher acuity, greater physiological instability, and more frequent secondary complications.

## 5 Conclusion

The sampled cohort performed marginally outside the predicted risk for mortality and functional outcome when assessed using the original SAHIT model coefficients, derived from a large international cohort of aSAH patients. With AUCs of 0.68–0.75 and wide CIs approaching 0.5, the SAHIT models fail to meet the minimum discrimination threshold (AUC > 0.80) required for individual clinical decision-making.^8^ Our findings support SAHIT’s utility for cohort benchmarking and research enrichment but not for individual prognostication in ICU settings. The significance of this deviation must be interpreted in the context of key differences between the study cohort and the original SAHIT repository. Factors such as a higher proportion of critically ill patients, variations in geographical and temporal practices, and differences in case selection methodology may have contributed to the observed drift in model accuracy.

`Despite these limitations, the SAHIT models hold potential for application in both unit performance assessment and early prognostication, offering insights into patient outcomes even before complications and confounding variables emerge. However, caution is necessary when interpreting results, particularly in cohorts with greater illness severity, as the accuracy of predictions may diminish under such conditions.

## CRediT authorship contribution statement

The authors would like to pay special thanks to Dr Nathan Jeffrey for his efforts in data collection and corroboration of patient outcome; Dr Nivene Saad for advice regarding CT interpretation; Jason Myers for data collection.

David Mogg is the corresponding author, davidwmogg@gmail.com; Adult ICU Mater Health Services Salmon Building South Brisbane, 4000, Brisbane, Queensland; RFDS Queensland Section.

## Funding

This research did not receive any specific grant from funding agencies in the public, commercial, or not-for-profit sectors.

## Conflict of interest

The authors do not have any conflict of interest to declare.
