# Supplementary material for: Assessment of outcomes in postaneurysmal subarachnoid bleed patients admitted to the intensive care unit utilizing the subarachnoid haemorrhage international trialist clinicoradiological prediction model for dichotomised functional outcome and mortality
Source: Crit Care Resusc. 2025 Oct 22;27(4):100126. doi: 10.1016/j.ccrj.2025.100126 (PMC12581637; doi:10.1016/j.ccrj.2025.100126)
Supplement: Multimedia component 1 [file mmc1.docx]

**Supplemental Appendix**

The SAHIT (Subarachnoid Haemorrhage International Trialists) prediction models are logistic regression-based tools used to estimate the probability of mortality and unfavourable outcomes in patients with subarachnoid haemorrhage (SAH). The models were developed using 10,936 patients and externally validated in six different studies^1^.

The SAHIT models incorporate three main levels of predictors:

1. Core Model – Includes patient age, history of hypertension, and World Federation of Neurosurgical Societies (WFNS) grade.
2. Neuroimaging Model – Extends the Core Model by adding SAH clot volume, aneurysm size, and location.
3. Full Model – Extends the Neuroimaging Model by including the treatment modality (surgical/clipping, endovascular/coiling, or none).

SAHIT Model Performance

1. Mortality Prediction:
   - Core Model: AUC = 0.76
   - Neuroimaging Model: AUC = 0.77
   - Full Model: AUC = 0.78
2. Unfavourable Outcome Prediction:
   - Core Model: AUC = 0.80
   - Neuroimaging Model: AUC = 0.81
   - Full Model: AUC = 0.81

The models showed good calibration across different cohorts and settings; however, calibration metrics are not reported.

**Table (a) Appendix: SAHIT Model Coefficients and Intercepts^1^**

| **Predictor** | **Mortality (Core)** | **Mortality (Neuroimaging)** | **Mortality (Full)** | **Unfavorable Outcome (Core)** | **Unfavorable Outcome (Neuroimaging)** | **Unfavorable Outcome (Full)** |
| --- | --- | --- | --- | --- | --- | --- |
| **Intercept** | -4.918 | -5.475 | -5.350 | -3.703 | -4.175 | -4.122 |
| **Age (per 10 years)** | 0.32 | 0.30 | 0.27 | 0.034 | 0.032 | 0.031 |
| **Hypertension** | 0.327 | 0.346 | 0.344 | 0.268 | 0.277 | 0.273 |
| **WFNS Grade 2** | 0.707 | 0.676 | 0.687 | 0.688 | 0.602 | 0.598 |
| **WFNS Grade 3** | 1.393 | 1.352 | 1.273 | 1.448 | 1.360 | 1.321 |
| **WFNS Grade 4** | 1.803 | 1.699 | 1.669 | 1.723 | 1.600 | 1.580 |
| **WFNS Grade 5** | 2.786 | 2.578 | 2.404 | 2.565 | 2.399 | 2.300 |
| **Fisher Grade 2** | — | -0.008 | 0.072 | — | 0.310 | 0.349 |
| **Fisher Grade 3** | — | 0.470 | 0.497 | — | 0.729 | 0.750 |
| **Fisher Grade 4** | — | 0.323 | 0.487 | — | 0.854 | 0.931 |
| **Location - ICA** | — | 0.220 | 0.222 | — | -0.105 | -0.109 |
| **Location - MCA** | — | -0.100 | -0.027 | — | -0.266 | -0.247 |
| **Location - PCA** | — | 0.473 | 0.318 | — | 0.032 | -0.033 |
| **Size 13-24 mm** | — | 0.658 | 0.481 | — | 0.222 | 0.136 |
| **Size ≥25 mm** | — | 1.178 | 0.370 | — | 0.529 | 0.131 |
| **Treatment - EVR** | — | — | -0.390 | — | — | -0.177 |
| **Treatment - None** | — | — | 1.543 | — | — | 0.842 |
| **Treatment - Surgical** | — | — | — | — | — | — |
